# Review on Polymer, Ceramic and Composite Materials for CAD/CAM Indirect Restorations in Dentistry—Application, Mechanical Characteristics and Comparison

**DOI:** 10.3390/ma14071592

**Published:** 2021-03-24

**Authors:** Aleksandra Skorulska, Paweł Piszko, Zbigniew Rybak, Maria Szymonowicz, Maciej Dobrzyński

**Affiliations:** 1 Department of Pediatric Dentistry and Preclinical Dentistry, Wroclaw Medical University, Krakowska 26, 50-425 Wrocław, Poland; maciej.dobrzynski@umed.wroc.pl; 2 Polymer Engineering and Technology Division, Wrocław University of Science and Technology, Wyb. Wyspiańskiego 27, 50-370 Wrocław, Poland; 3 Department of Experimental Surgery and Biomaterials Research, Wroclaw Medical University, Ulica Bujwida 44, 50-368 Wrocław, Poland; zbigniew.rybak@umed.wroc.pl (Z.R.); maria.szymonowicz@umed.wroc.pl (M.S.)

**Keywords:** CAD/CAM, mechanical properties, dental materials, dental ceramics, resin composites, biocompatibility

## Abstract

The aim of this review article is to present various material groups, including ceramics, composites and hybrid materials, currently utilized in the field of CAD/CAM. The described technology is amongst the most important in modern prosthetics. Materials that are applicable in this technique are constantly tested, evaluated and improved. Nowadays, research on dental materials is carried out in order to meet the increasing demand on highly aesthetic and functional indirect restorations. Recent studies present the long-term clinical success of restorations made with the help of both ceramic and composite materials in the CAD/CAM method. However, new materials are developed and introduced that do not have long-term in vivo observations. We can outline a monolithic polymer-infiltrated ceramic network and zirconia teeth support that show promising results to date but require further assessment. The materials will be compared with regard to their mechanical and clinical properties, purpose, advantages and limitations.

## 1. Introduction

CAD/CAM stands for computer-aided design and computer-aided manufacturing. It is applied in different branches of engineering, science or even art as a rapid prototyping method for accelerating the design process and smoothening its transition into manufacturing [1]. In this review article, we focus on its constantly growing role in dentistry, more precisely, in dental prosthetics. This field of dentistry serves for the manufacturing of either polymer, ceramics or composite teeth restoration. CAD/CAM allows us to provide a patient with dental prosthesis, such as crowns, veneers, bridges, inlays, onlays and dental implant supported restorations [2,3,4]. In many cases, due to the technology used in CAD/CAM, it is possible to scan a patient’s oral cavity, design and create restoration and finally bond it in the patient’s mouth in the course of one day. There are both chairside systems and systems which further outsource the prosthesis manufacturing available on the market. To obtain the required properties of restorations, such as high aesthetics, biocompatibility, durability and functionality, a wide range of constantly evolving materials are used in the system described below [5].

## 2. Materials and Methods

An electronic search of the English-language and Polish-language literature published between 1987 and August 31, 2020, was conducted within PubMed, Scopus, Web of Science and Google Scholar databases. A combination of free-text words was used: CAD/CAM, ceramics, resin composites, dental material, methacrylates, biocompatibility, cytotoxicity and chairside systems. A group of articles was outlined for further verification with respect to their contribution to the topic. Authors focused on articles regarding polymer, ceramic and composite dental CAD/CAM materials along with their mechanical characterization, wearing, cytotoxicity and clinical assessment. Afterwards, the reference list was selected and narrowed down in the scope of the relevance in the field of intraoral CAD/CAM materials utilized in dentistry and their characteristics.

## 3. Historical Outlook on CAD/CAM

The first versions of the system were invented in the 1980s [6]. The idea of the digital assisted prosthetic system was first developed and introduced as a result of the cooperation of three research centres, the first established by the University of Zurich and Brains and Brandestini Instruments of Switzerland, the second by Hennson International of France and the third group by the University of Minnesota [7]. The aim was to provide the patient with a prosthetic restoration in a fast and impressionless process. Moreover, the authors intended to make durable posterior teeth restorations in a natural colour [6]. Recent years have shown that it is an innovative, developing and forward-thinking method of designing and forming dental prostheses.

## 4. Advantages of CAD/CAM

The individual properties of CAD/CAM restorations depend, among others, on material which is used for manufacturing. There is wide range of benefits shared by all used materials, which make described technology very attractive both for the patient and dentist. Among them, we can list the following: shorter time of prosthetic treatment; patient’s satisfaction; and avoiding the traditional method of making impressions, which has been replaced with user-friendly intraoral scanners [8,9]. There are randomised clinical trials that confirm higher efficiency and comfort using digital scanning compared to conventional impression [10,11]. Moreover, it provides the opportunity to combine high aesthetics, durability and functionality in one restoration [12,13]. The aspect of accuracy is very important as well. It was confirmed that the CAD/CAM restorations, such as single crowns, fixed dental prostheses and implant-retained fixed dental prostheses, are characterized by sufficient marginal adaptation [14,15]. This fact is significant for further plaque accumulation. Potential caries development is less likely to occur when the marginal adaptation is within the clinically acceptable marginal discrepancy range. Replacing the classical procedure of taking impressions by the digital technique helps not only to reduce procedure time [16] and increase the patient’s positive feelings but also to maintain an adequate level of precision (4 to 80 μm for scans with a limited area) [17]. Furthermore, scanning oral cavity by an intraoral scanner provides an image of prosthetic substrate on the computer screen almost immediately and under magnification, which helps to control the preparation process as well as plan further restoration. This is an important benefit for many operators. The technology provides the opportunity to use new materials for prosthetic reconstruction and maintain the quality control of the process [18]. All these positive aspects of CAD/CAM technology are reflected in patients’ satisfaction and long-term restoration success considering both ceramic [19,20] and composite restorations [21,22].

## 5. Limitations and Handling of the System

The technology of CAD/CAM, without a doubt, is very innovative and provides a broad range of opportunities. However, this technique is still considered expensive, and despite the development of new systems and increasing competition on the market, the prices remain high [2,23]. The whole process of creating a restoration using CAD/CAM comprises many steps, such as scanning the oral cavity by an intraoral scanner, computer designing using specific software and modelling a restoration either from a solid block of restorative material or using an additive technique [23]. All of this requires highly trained personnel [2], and the technique learning curve can range from a few days to several months [24]. Moreover, as opposed to the traditional way of planning prosthetic reconstruction, in the CAD/CAM system, the involvement of the patient is minimalised [25]. After scanning the oral cavity, the dentist decides the shade, shape and occlusal relation of the prosthetic restoration. Considering clinical cases regarding patients with maxilla-mandibular disorders and occlusion distortions, the CAD/CAM system itself may not be sufficient to obtain correct teeth relation [26]. Moreover, the size of the blocks limits designing and milling restorations exceeding their sizes. This indicates clinical problems including inaccurate occlusal vertical dimension and incorrect centric relation [27].

The accuracy of digital scans depends on the length of the arch included in the impression and is favourable for short distances [28]. The survival rate of CAD/CAM restorations may vary for different types of materials. It is mostly presented in short- and medium-term studies, which makes it more difficult to evaluate and compare to conventional prosthetic restorations. For example, ceramic material Vita Mark II (VITA Zahnfabrik, Bad Säckingen, Germany) inlays showed survival rates of 90.6% after 8 years and 85.7–89% after 10 years [13,29,30,31]. Therefore, we observe that survival rate decreases over time. The mechanical aspects, such as the flexural strength or mean modulus of resilience, differ for various types of utilised materials [32]. It can be assumed that not all kinds of materials are suitable for all clinical applications. The prosthetic restorations made using CAD/CAM are not free of defects. The main reported complications are framework fractures and recurrent periodontal disease for reinforced glass ceramics and glass infiltrated alumina [16]. Thus, there is still room for improvements in the described technique.

Operators should be aware of certain limitations regarding patients with CAD/CAM restorations. For example, applying lasers for periodontal or conservative reasons among patients with zirconia-based restorations can be performed. It should be considered that the surface of the restorative material can be affected by the laser [33]. Moreover, mechanical limitations are of significant importance, which should not be omitted while describing CAD/CAM restorations. The study by Romanyk et al. shows that subtractive machining results in strength-limiting, surface and subsurface damage in the restorations, which may be clinically relevant [34].

## 6. Currently Used Dental CAD/CAM Systems

The list of producers offering CAD/CAM software (e.g. CEREC SW 5.1.3, Dentsply Sirona, York, Pennsylvania, United States) and manufacturing systems is broad and has rapidly grown in recent years. CAD/CAM systems can be classified as either in-office or laboratory systems [35]. The two most popular systems currently available on the market are CEREC by Dentsply Sirona (York, PA, USA) and Planmeca by Planmeca Oy (Helsinki, Finland) [36]. Both of them are complex and consist of numerous elements. For example, Sirona offers the CEREC Omnicam scanner, software for CAD/design and for CAM and also the milling unit, which is the CEREC MC, X and XL 4-axis milling machine [8]. Other recognized and used total CAD/CAM systems are Carestream Dental (Atlanta, GA, USA), Dental Wings (Montreal, QC, Canada) and Zfx (Dachau, Germany) [36]. There is also the possibility to buy parts included in a CAD/CAM system, which are offered separately by different companies. The choice of adequate system depends on the prosthetic experience of operators and the equipment of the dental office, but should be also dictated by the patient’s therapeutic needs [37].

## 7. Computer-Aided Design 

After scanning the oral cavity using an intraoral scanner, such as powder-free CS 3600 by Carestream Dental [8], we are able to obtain a digital image of the oral cavity, which is the field for further prosthetic restoration. We can divide scanners into two groups: those that require powder and powder-free. Powder scanners require an opaque reflective coating, such as titanium dioxide powder, on the teeth before scanning in order to eliminate reflection and to create an equal surface. On the other hand, powder coating may reduce the scan precision and marginal adaptation of definitive restoration. [38]. Nowadays, mostly powder-free scanners, such as CS 3600 by Carestream Dental (Atlanta, GA, USA), are used [8]. In this group of scanners, there is no risk of mixing intraoral liquids and powder, and thus blurring the boundaries of the preparation. The digital impression is required in multiple steps of the restorative process, including preliminary scanning for the clean-up process followed by the margin setup by the technician before the fabrication takes place. Therefore, they are not considered to save time in comparison to the conventional impressions [39]. Moreover, the resolution of full arch digital impressions is limited in comparison to the conventional impressions [40].

Appropriate software helps to design a restoration in the most optimal way, allowing the operator to make changes simultaneously if needed. In the case of Carestream Dental, a dedicated software for the system is CS Restore. Plenty of different software is available on the market, such as DWOS Chairside (Dental Wings), Cerec SW 4.5. and Cerec Premium SW 4.5 (Dentsplay Sirona), Zfx CAD software (Zfx), Planmeca PlanCAD Easy, integrated in Planmeca Romexis (Planmeca) or MyCrown Design (Fona Dental) [8].

## 8. Materials for CAD/CAM

The spectrum of materials utilized in computer-aided manufacturing is very broad. It includes not only acrylics polymers but multiple ceramic materials and resin composites [41]. Moreover, the conventionally utilized restoration materials for dental prosthetics include metals. However, due to the necessity of ceramic veneering, we are no longer in the scope of chairside CAD/CAM due to the required postprocessing [42]. Thus, in this review, metals are not covered, and the main focus is put on polymer, ceramic and composite materials. 

**Figure 1:**
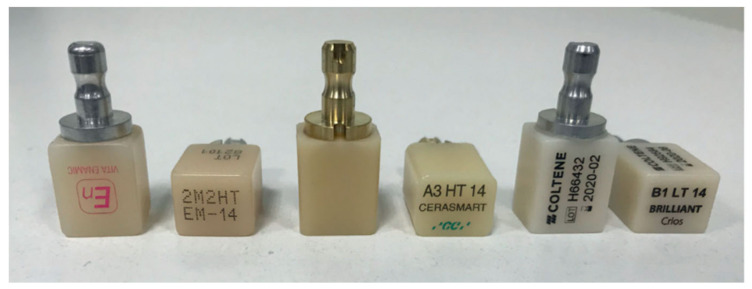
Examples of commercial CAD/CAM blocks. From the left: resin matrix composite Vita Enamic, resin matrix composite CERASMART and composite Brilliant Crios, reprinted with permission from ref. [44] (Copyright 2020 Inżynier i Fizyk Medyczny).

Each material has different processing parameters, and, therefore, the whole system needs to be adjusted for a specific material. The success of prosthetic treatment using CAD/CAM technology depends, to a certain extent, on material selection but also on all steps of a treatment: from case classification, to correct preparation, precise scanning, planning and designing, resulting in the manufacturing and cementing of a restoration. Along with the desirable characteristics of materials used for restorations in the chairside procedure, the efficiency lies in the possibility of high-speed milling without damage and a short time of preparation of the restoration after milling [43]. The examples of CAD/CAM blocks before processing are depicted in Figure 1 (below).

### 8.1. Dental Ceramics

**Figure 2:**
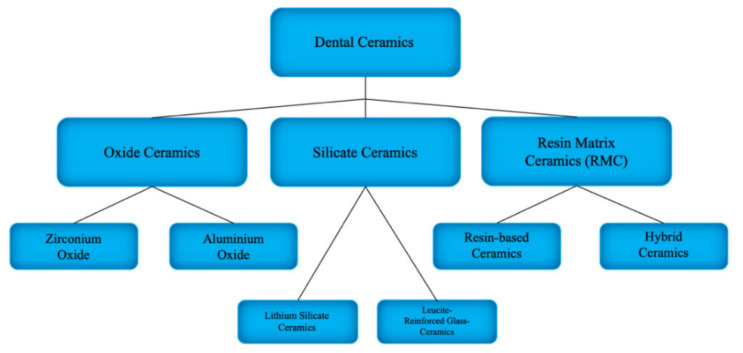
Dental ceramics classification.

There are various types of dental ceramics with respect to their chemical composition, method of obtaining and structure. They can be classified into 3 groups: resin matrix ceramics (RMCs), silicate ceramics and oxide ceramics (see Figure 2) [43]. In general, ceramics can be characterized by strength, brittleness, transparency and hardness [17,45,46]. All of the ceramic restorations made using the CAD/CAM system can be used both in posterior and aesthetic segments and are becoming increasingly popular every year. Their main advantages include biocompatibility, low plaque adherence susceptibility and colour stability [17].

Ceramics are crystalline and non-metallic materials, while glass ceramics are composite-type materials in which the glassy phase is the matrix and the ceramic is the reinforcing filler [43]. All-ceramic CAReviD/CAM restorations demand a rounded shoulder or a heavy chamfer around the circumference of the prepared tooth. In most cases, luting using adhesive resin cements is indicated for all-ceramic crowns. This helps to increase fracture resistance [47]. The outline of the ceramics division is presented in Figure 1. Each of the enlisted ceramic type has a different clinical application due to its properties (see Table 1).

#### 8.1.1. Resin Matrix Ceramics

This is a relatively new material to the market, but it is claimed that it shows some favourable characteristics for dental prosthetics. Resin matrix ceramics are characterized by lucrative milling properties and, compared with silica-based ceramics, have a higher load capacity and better modulus of elasticity [36]. The aesthetic aspect is also satisfying for resin matrix ceramics, which shows optical properties similar to natural teeth.

We can distinguish resin-based ceramics (e.g., Lava Ultimate by 3M-ESPE, Seefeld, Germany) that contain a polymer matrix with at least 80% nanosized ceramic filler particles and hybrid ceramics (e.g., VITA Enamic by VITA-Zahnfabrik, Bad Säckingen, Germany and Cerasmart by GC, Leuven, Belgium) made of a ceramic network infiltrated with a polymer using polymer-infiltrated ceramic network (PICN) technology [36,44]. Recent studies report that resin-based ceramics show flexural strengths up to 230 MPa, characteristic strength (σ_0_: 300 MPa) and relatively low Young’s modulus [48]. The manufacture of Lava Ultimate characterizes this material as less brittle than glass ceramic and resistant to chipping and cracking when milled [49]. VITA Hybrid ceramics combine the properties of both composites and ceramics, which leads to sufficient flexibility, optimal distribution of chewing forces and high resistance to loads [17]. The manufacture process of VITA Enamic ensures that the tendency of fracturing is lower in comparison with pure ceramics, and CAD/CAM processing is superior [50]. Moreover, their optical properties are similar to natural teeth, and they are characterized by lower abrasion for opposing teeth compared to ceramics [44].

#### 8.1.2. Silicate Ceramics

These are non-metallic inorganic ceramic materials that contain a glass phase. Among silica-based ceramics, we can distinguish feldspathic and lithium silicate ceramics. Examples are Vitablocs TriLuxe by Vita and IPS Empress CAD Multi by IvoclarVivadent. They can be characterized by favourable optical aspects, such as high translucency and natural appearance. However, the presence of glass in their compositions contributes to the brittleness and low fracture strength [36]. Silicate ceramics require hydrofluoric (HF) acid etching to enhance micromechanical retention [51] and adhesive bonding. After acid etching, the glassy matrix is dissolved and crystalline phase is exposed, and thus, the surface of the ceramic becomes available for the micromechanical interlocking of resin cement [52]. The study evaluating tensile bond strength for lithium disilicate ceramic confirms that etching the bonding surface of restorations with hydrofluoric acid is still a “gold standard” [53].

#### 8.1.3. Leucite-Reinforced Glass Ceramics

The clinical long-term valuation of leucite-reinforced glass restoration ceramics (e.g., Duraceram and Dentsply Degussa) was discussed. Leucite-reinforced ceramics are not recommended for crowns in the posterior segment due to their lower mechanical properties compared to other glass ceramics [54]. However, their aesthetic qualities are sufficient, and the wear resistance of the enamel antagonist is similar to other glass ceramic materials [55]. In recent years, they have been replaced by lithium silicate ceramics, which have better physical properties and sufficient optical properties.

#### 8.1.4. Lithium Silicate Ceramics

Some sources claim that lithium silicate ceramics (e.g., IPS e.max CAD by Ivoclar Vivadent, Schaan, Liechtenstein, VITA Suprinity PC by VITA Zahnfabrik and Celtra Duo by Dentsply Sirona) are the strongest of all the available silicate ceramics with a flexural strength of around 407 MPa [36]. First, lithium disilicate ceramic was introduced to the market in 1998 (IPS Empress 2) [56]. Its chemical composition—a crystalline phase consisting of lithium disilicate and lithium orthophosphate—indicates higher fracture resistance without a negative influence on the translucency of the material [36,57,58].

It shows good clinical results in follow-ups with a failure-free rate at the level of 93% [59]. There is also a study which indicates its superior colour stability in different staining solutions, such as coffee or cola, compared to high-translucency zirconia, nanoceramic or hybrid ceramic [60].

#### 8.1.5. Oxide Ceramics

Oxide ceramics exhibit highly favourable mechanical properties, while their aesthetic qualities are slightly worse than silicate ceramics due to their low translucency. They can be divided into aluminium-oxide- and zirconium-oxide-based ceramics.

The oxide ceramics are divided into two major groups:

• Aluminium oxide ceramics

Described as glass-infiltrated aluminium oxide core ceramics (InCeram Alumina, VITA Zahnfabrik, Bad Säckingen, Germany), they are characterised by a flexural strength of 500 Mpa. They show satisfying results in long-term follow-ups, notwithstanding in recent years, when they have mostly been replaced by more popular zirconia ceramics characterized by superior physical properties [61].

• Zirconium oxide ceramics

**Figure 3:**
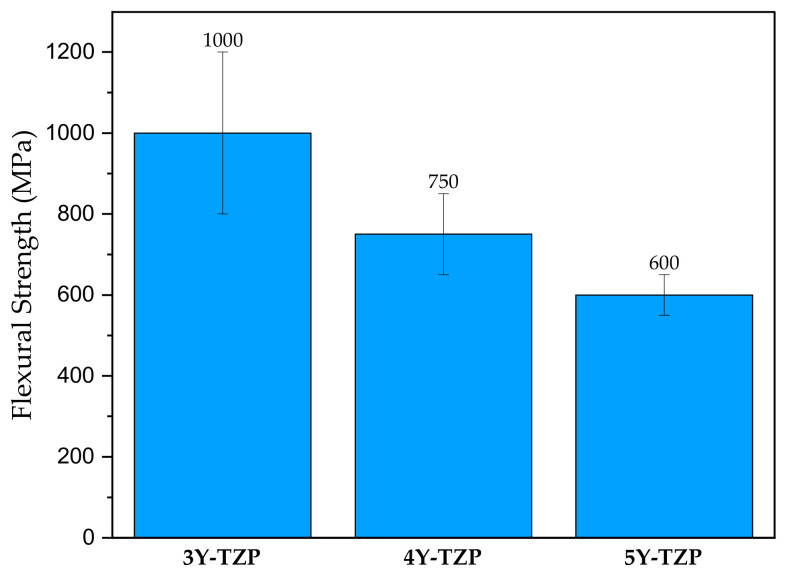
Flexural strength IPS e.max ZirCAD zirconium oxide ceramics varying in yttrium oxide content: 3%-3Y-TZP, 4%-4Y-TZP and 5%-5Y-TZP Adapted from ref. [64].

Commercial CAD/CAM zirconium oxide ceramics are present in the form of yttria-stabilized tetragonal zirconia polycrystal (Y-TZP) [62,63] in products such as Lava Plus (3M, ESPE) or Kavo Everest (KaVo Dental). In the product range of IPS e.max ZirCAD (Ivoclar Vivadent), we can find Y-TZP stabiliized by a 3,4 or 5% addition of yttrium oxide (3Y-TZP, 4Y-TZP or 5Y-TZP). The mechanical properties are dependent on the chemical composition. For example, the flexural strength of ZirCad products decreases with the addition of yttrium oxide [64] (see Figure 3). For a long-term durable bond, a complex protocol is recommended by a producer [36]. Tooth-Supported Zirconia Single Crowns show good results in long-term in vivo observations [65] and general biocompatibility [66].

### 8.2. Polymer-Based Materials

**Figure 4:**
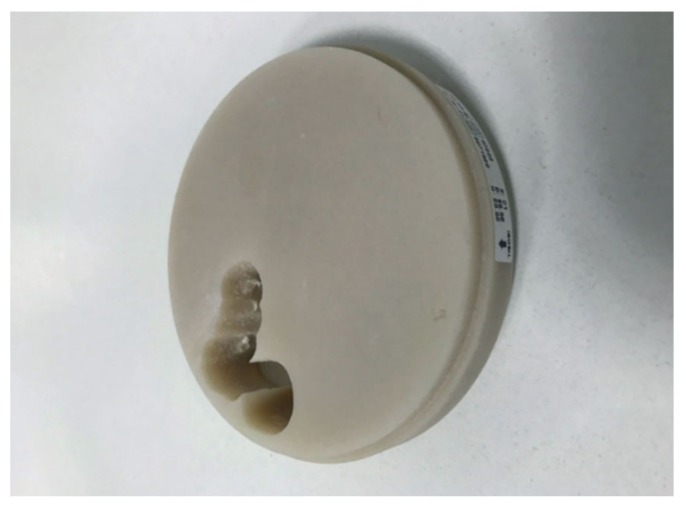
PMMA disk, reprinted with permission from ref. [44] (Copyright 2020 Inżynier i Fizyk Medyczny).

Due to its mechanical properties and biocompatibility, poly(methyl methacrylate) (PMMA) was introduced as a CAD/CAM material (see Figure 4) for manufacturing protheses [67,68]. Moreover, PMMA resin is among the oldest acrylic materials used in dentistry. The PMMA in CAD/CAM blocks occurs in cross-linked form (eg. Telio CAD, Ivoclar Vivadent), unlike conventional dental application where it is subjected to photocuring. The highly cross-linked nature of those materials puts them before conventionally polymerized interim resins in terms of durability and processing ease [69].

**Figure 5:**
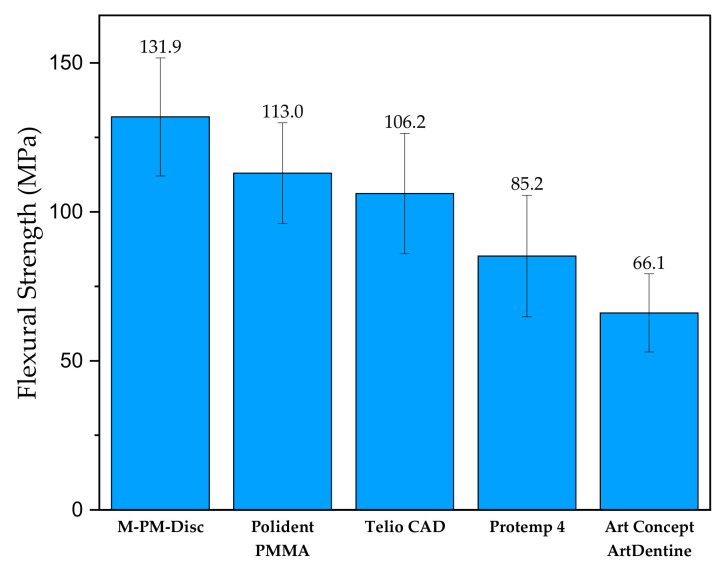
Flexural strength of different commercial interim PMMA-based resins. Adapted from ref. [68].

As stated in Alp et al. (2019), the CAD/CAM PMMA-based polymers exhibited greater flexural strength than bis-acrylate derivatives as well as conventional PMMA interim resin material [68]. Figure 5 (below) clearly shows that three CAD/CAM PMMA-based materials (M-PM-DISC, Polident PMMA and Telio CAD) exhibit greater flexural strength than Protemp 4 (Bis-acrylate composite resin) or Art Concept ArtDentine, which is conventional PMMA. During the storage of the prepolymerized PMMA-based polymer, the relaxation of the material occurs.

Among the other high-performance polymers utilized in the CAD/CAM systems for manufacturing we poly(ether ether ketone), commonly known as PEEK. Like many other polymeric materials used in dentistry, it has thermoplastic characteristics and increased biocompatibility. It is worth mentioning that PEEK has an elasticity modulus at the level of 3–4 GPa [70], which is very close to the modulus of Type 3 Spongeous bone [71]. This polymer was concluded to be applicable for removable prosthetics due to its mechanical properties, low discoloration and minimal monomer content when produced by means of CAD/CAM [72,73].

PMMA-based materials have been suggested as a long-term interim prosthesis [74,75,76]. By definition, this material should have adequate mechanical resistance to withstand the mechanical forces of the jaw during teeth collision.

### 8.3. Composites

With the development of CAD/CAM materials, and increasing doctor and patient expectations, hybrid structures, such as composite resins, hybrid ceramics or conventional materials with additives altering their physical properties, have been introduced to the market. Composites are known to be materials made of at least two substances, exhibiting properties from each of them. It is no surprise that regarding CAD/CAM, an additive for polymeric or ceramic materials is used in order to enhance the tribological or mechanical properties or simply the aesthetics of the prostheses. For example, 0.3–0.5 µm ceramic particles are added to PEEK for property optimization [77]. The division of such composites is based on their structure and manufacturing process. We can outline polymer-infiltrated ceramic networks (PICN) and resin composite blocks (RCB). The former consists of two phases: a ceramic scaffold and an interpenetrating polymeric network [42]. The latter are formed by transferring the filler into a monomer mixture [78].

**Figure 6:**
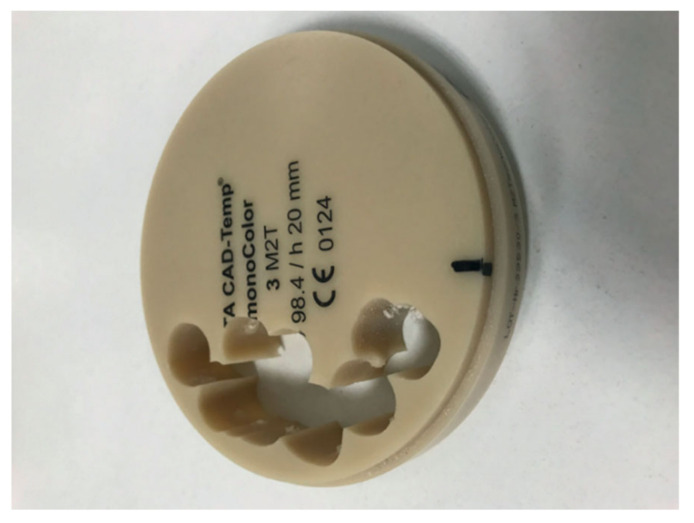
Vita CAD-Temp disk, reprinted with permission from ref. [44] (Copyright 2020 Inżynier i Fizyk Medyczny).

A study on the resistance of resin composite crowns was performed using the example of the maxillary first molar, and lithium disilicate glass ceramic (IPS e.max CAD by Ivoclar vivadent) was utilized as a control reference [79]. In the same work, an evaluation of fracture strength and biaxial flexural strength showed satisfactory results. All CAD/CAM composite blocks that were tested (among them Shofu block HC by Shofu Inc., KZR-CAD HR by Yamamoto precious metal Co. and Katana avencia by Kuraray noritake dental) exhibited about 3–4 times higher fracture strength than the average maximum bite force of the molar tooth (700–900 N). An example of a composite CAD/CAM disk is presented in Figure 6 (below). 

It is crucial to mention that dental composites vary in properties depending on composition. A comprehensive study conducted by Stawarczyk et al. [76] evaluated the mechanical as well as optical behaviour of commercial composites. The research concluded that regarding flexural strength, CAD/CAM composites generally exhibit higher flexural strength over leucite ceramic material, but lower than lithium silicate ceramic. The same study stated that glass ceramics showed a lower discoloration rate in comparison with CAD/CAM composites.

## 9. Nanoscale Aspect of CAD/CAM Materials

Nanotechnology has a wide range of applications in medicine and dentistry. By incorporating nanomaterials, one can alter the properties of different materials. It is possible to enhance the optical, chemical, electrical and mechanical properties of materials by adding suitable nanoparticles [80,81]. Nanosized filler particles are added to the resin-based composites to fill the space between larger filler particles and at the same time reduce the content of the resin [82].

When considering nanoparticles, safety is of vital importance. The intraoral CAD/CAM restorations as well as other dental materials are subjected to processes of wearing and degradation over time. Therefore, the issue of the release of nanoparticles over time and their possible negative effect on the organism needs to be addressed [83]. When scaling down to nanosized particles, we observe an increase in the surface area, which enhances their chemical activity in the scope of potential interactions inducing an adverse cellular response [84].

Among the materials utilized in CAD/CAM technology, we can outline resin matrix ceramics, also called nanoceramics [48]. This material was invented in order to meet the need of combining the ceramics’ high aesthetic and mechanical advantages of composites. The examples are Cerasmart, GC Dental Products and Lava Ultimate, 3M ESPE, which owes its beneficial properties to nanotechnology used to bind nanoceramic particles into a resin matrix [49]. The producer describes its composition as 80% nanoceramic and 20% resin matrix [85] (average particles size: 20 nm for silica particles and 4–11 nm for zirconia particles) [60], while Ceramsmart is composed of 71 wt.% silica and barium glass nanoparticles [52]. Nanoceramics are considered optimal materials for restorations in aesthetic segments. Their high translucency is an effect of nanosized zirconia and silica particles that decrease light scattering [52,86]. Moreover, the addition of nanoparticles to composite resins improves the tensile and compressive strength, which contributes to reducing secondary caries by the elimination of microleakage [80].

Whereas nanotechnology in dentistry provides a wide array of possibilities in modifying the properties of the material (enhancing tribological and mechanical properties, lowering cytotoxicity), it is necessary that researchers and clinicians take into consideration its long-term effect and potential toxicity. Although nanoparticle addition is generally considered not to cause a negative toxicological response, both beneficial and adverse implications of nanotechnology in dentistry are thoroughly covered by R.N. Al Kahtani et al. [87].

## 10. Comparison of CAD/CAM Materials

Both resin composite and ceramic CAD/CAM materials have advantages and drawbacks for intraoral application. It is of vital importance to select the correct material depending on the patient’s personal needs and considering mechanical and visual material characteristics. Resin composites are attractive because of their machinability and intraoral reparability, while glass ceramics/ceramics may offer superior mechanical and aesthetic properties [43].

The final dental restoration or prosthesis should resist occlusal forces that appear during the clinical service [68]. Moreover, the mechanical parameters of conventional interim materials change over time and are affected by wearing and their chemical characteristics (e.g., susceptible for water sorption [88]). We can outline multiple mechanical properties characterizing CAD/CAM materials: flexural strength, fatigue stress resistance, hardness and elastic modulus in addition to optical properties such as colour and translucency. Every major CAD/CAM material group is presented in the Table 2, along with their flexural strength, hardness, elastic modulus and composition.

Flexural strength is an important property giving an idea of the general mechanical strength and rigidity of the presented material with respect to dental application prosthesis [89]. This might lead to the conclusion that the higher the value is, the better. High flexural strength is essential to successful clinical procedure, but one must take into consideration other mechanical properties depending on the final application, i.e., onlay, inlay, crown, bridge or arch. Among those with the highest flexural strength, we can outline IPS e.max zirCAD (zirconium oxide ceramics, Ivoclar Vivadent, Schaan, Liechtenstein) with 850 MPa or VITA In-Ceram ALUMINA (aluminium oxide ceramics, VITA Zahnfabrik, Bad Säckingen, Germany) with 419 MPa. On the other hand, there are materials that exhibit very low flexural strength, such as VITA CAD-Temp (composite, VITA Zahnfabrik, Bad Säckingen, Germany) with 80 MPa or VITA ENAMIC (hybrid ceramic, VITA Zahnfabrik, Bad Säckingen, Germany) with 150–160 MPa.

The listed parameters clearly show the fluctuations in their hardness values for every type, e.g., aluminium oxide ceramics have 2035 HV hardness, while PMMA has just 26 HV—approximately two orders of magnitude more. Hardness is essential with regard to material wearing and scratching resistance. Hence, the ideal enamel replacement material would have similar hardness to the human teeth tissues, which is approximately 274.8 ± 18.1 HV [90]. We can see that the VITA ENAMIC hybrid ceramic exhibits a value of 200 HV, which indicates that it is a potential candidate for enamel substitution.

Materials are subjected to deformation after load. Those with a low elasticity modulus will be more strongly deformed than materials with a higher modulus [91]. This, yet again, indicates the necessity for clinical application. When mechanical load bearing or teeth mechanical collision is present, a restoration wears at a faster rate. A dentist might consider a material with a higher elastic modulus, such as VITA In-Ceram ALUMINA (aluminium oxide ceramics, VITA Zahnfabrik, Bad Säckingen, Germany) with 410 GPa [92] or IPS e.max CAD (lithium silicate ceramic, Ivoclar Vivadent, Schaan, Liechtenstein) with 103 GPa [93] for a longer lifespan of the restoration. As it is shown, every material has its weak points and advantages, and, therefore, the dentistry specialist needs to bear this in mind while selecting the right material for the purpose.

Fatigue stress has a significant influence on degradation and the material’s fracture response. Mechanical degradation and water-assisted corrosion lead to a reduction in the stress intensity threshold for fracture initiation, as concluded in a comprehensive study on 8 commercial CAD/CAM materials. Moreover, the study gave an insight into their lifetime predictions, showcasing the maximum applied stress as the percentage of characteristic strength [94].

The advantage of CAD/CAM materials over the direct fabrication technique was confirmed in a study where fixed partial dentures made from three interim resin materials were stored in different conditions for 5000 thermocycles. The study concluded that the flexural strength of acrylate-based CAD/CAM-fabricated fixed partial dentures (Luxatemp AM Plus, Carcon Base PMMA, Trim) was greater than that of those manufactured from the same materials in a direct manner [67]. Although the fabrication method affected the maximum force at fracture values of CAD/CAM and directly fabricated fixed partial dentures, the scanning electron microscopy analysis did not show any porosities or voids that may affect the overall strength among specimens. Therefore, this could be explained by the higher load bearing capacity of the polymeric phase manufactured by means of CAD/CAM. Moreover, as we observe a major development in the field of nanomaterials and complexity in material composition (see Table 2), in the near future, the technology will allow for manufacturing additives for dental materials which will affect the mechanical properties in a selective manner for a tailored-fit CAD/CAM block. 

**Table 2:** Mechanical properties and chemical composition of selected CAD/CAM materials.

Material Type	Product, Manufacturer	Flexural Strength (MPa)	Hardness (HV)	Elastic Modulus (GPa)	Composition	References
**Composites**	VITA CAD-Temp, Vita Zahnfabrik	80	n/a	2.8	Acrylate polymer with microparticle filler	[44,95]
**Aluminium oxide ceramics**	VITA In-Ceram ALUMINA, Vita Zahnfabrik	419	2035	410	Al_2_O_3_ (82 wt.%), La_2_O_3_ (12 wt.%), SiO_2_ (4.5 wt.%), CaO (0.8 wt.%), other oxides (0.7 wt.%)	[96]
**Zirconium oxide ceramics**	IPS e.max zirCAD, Ivoclar Vivadent	1200	n/a	206.3	3 mol% Yttria-stabilized tetragonal zirconia polycrystals (3Y-TZP)	[93,97]
**Lithium silicate ceramics**	IPS e.max CAD, Ivoclar Vivadent	353.1	617	102.7	SiO_2_, Li_2_O, K_2_O, P_2_O_5_, SiO_2_, ZnO	[85,91,98,99]
**Leucite-Reinforced Glass Ceramics**	IPS Empress CAD, Ivoclar Vivadent	160	632.2	62	SiO_2_ (60–65 wt.%), Al_2_O_3_ (16–20 wt.%) K_2_O (10–14 wt.%) Na_2_O (3.5–6.5 wt.%), other oxides (0.5 wt.%), pigments	[100]
**Resin-based ceramics**	Lava Ultimate, 3M	200	96	12	Polymerizable resin, dispersed nanometric colloidal silica, ZrO_2_ spherical particles	[48,85,93,101]
**PMMA**	Polident PMMA, Polident	114	26	2.77	PMMA, pigment	[102]
**Hybrid cermics**	VITA ENAMIC, Vita Zahnfabrik	150–160	200	30	SiO_2_, Al_2_O_3_, Na_2_O, K_2_O, B_2_O_3_, ZrO_2_, CaO, urethane dimethylacrylate, triethylene glycol dimethylacrylate	[50,85]
**PEEK**	PEEK-OPTIMA™, Invibio	165	n/a	3.70	PEEK	[103,104,105]

## 11. Adhesion–Bonding of CAD/CAM Restoration 

Effective and stable bonding contributes to long-term high clinical success rates. Resin bonding and self-adhesive resin cements are vastly recommended for CAD/CAM restorations. A. Mine et al. created a review providing a broad outlook on the bonding procedures of CAD/CAM materials [106]. The study states that the bonding procedure should be preceded by generating microretentive surfaces by hydrofluoric acid etching. The next step of the procedure is silanization, the aim of which is ensuring chemical adhesion [107]. The study considers the presented bonding procedure regarding indirect resin composite materials (including Lava Ultimet, KATANA AVENCIA block, Gradia Block, Ceras- mart, Paradigm and Block HC) and CAD/CAM polymer-infiltrated ceramics (such as Vita Enamic) [107]. Authors of the aforementioned review also notice that it is possible to improve bonding to CAD/CAM PMMA resin materials by using materials containing MMA. These are general recommendations on bonding for most CAD/CAM materials, which may vary depending on the producer’s recommendations and personal experience of clinical operators. As presented in another study from 2018 [108], resin bonding has long been the gold standard for the retention and reinforcement of silica-based ceramics. However, due to the complexity of the bonding procedure compared to conventional cementation, many dentists seek a simplified bonding protocol. The bonding procedure from the technical and scientific documentation of Vita Enamic can be presented as an example [50]. Authors propose the following protocol: Etching for 60 s with VITA CERAMICS ETCH (5% hydrofluoric acid gel) then silanizing with VITASIL, VITA or Monobond Plus, Ivoclar Vivadent. The next step is using the bonding composites RelyX Unicem (3M, Seefeld, Germany) and Variolink II (Ivoclar Vivadent, Schaan, Lichtenstein) in accordance with the manufacturer’s instructions. This procedure ensures optimal bonding with compressive shear strength of approximately 20 MPa.

## 12. User Experience of CAD/CAM Hybrid Materials

In fact, VITA Enamic (VITA Zahnfabrik) seems to be an adequate material not only for crowns in the posterior segment but also in aesthetic segment or restorations with reduced wall thickness. This provides the possibility to create restorations based on implant frameworks. The structure of VITA Enamic is based on two interpenetrating networks—dominant ceramic mesh (86%) is reinforced by a polymer (14%). As mentioned, the structure of the hybrid material allows us to combine the beneficial properties of ceramics and composites. VITA Enamic has optimal flexibility, stress resistance and light conductivity, which provide better visual adaptation. To adjust the optical aspect, it is available in mono- and multichromatic types in three translucency levels. All these factors influence not only patient satisfaction but also make dentist work more comfortable. After designing, as an effect of milling, we can obtain anatomically precise restorations. It is worth mentioning that milling, which in our case was performed by the CEREC System, is faster (about 6.5 min for molar crown) for this material compared to others. According to the manufacturer, milling performed on Sirona MC XL (normal mode, Dentsply Sirona, New York City, NY, USA) for VITA Enamic blocks takes 7:56 min for the inlay, 7:10 min for the anterior crown and 9:07 for the posterior crown [50]. The next step after milling is the cutting of the sprue, polishing, preparing the inside surface by etching with hydrofluoric acid applying a silane and bonding agent and cementing using resin cement. To support the information provided by the manufacturer and our clinical experience, the study of flexural strength, flexural modulus, fracture strength and microhardness of different CAD/CAM materials may be referenced. Researchers compared IPS e.max CAD (lithium disilicate), VITA Suprinity (zirconia- reinforced lithium silicate), GC Cerasmart (hybrid high-performance polymer composite resin) and VITA Enamic. As a result of that study, all tested materials were considered as suitable for posterior full-crown restorations, but hybrid materials showed lower hardness and stiffness compared to glass ceramics [109]. Similar properties were presented in another study, which showed that the microhardness, flexural strength and fracture toughness of VITA Enamic are similar compared to IPS e.max CAD (Ivoclar Vivadent), IPS Empress CAD (Ivoclar Vivadent) and VITA Mark II (VITA Zahnfabrik) [110].

## 13. Surface Finishing and Disguise of CAD/CAM Restorations.

Polishing and glazing of CAD/CAM restorations as the finishing elements is crucial for smoothing the surface of the restoration and preserving the optical properties of the material. After milling the ceramic and composite resin blocks by diamond burs, the surface becomes rough. The roughness of the surface not only increases the level of biofilm accumulation [111,112], which may negatively affect gum tissues, but also facilitates discoloration [113,114]. There is also a number of mechanical consequences of surface roughness, such as decreasing mechanical resistance [115] or causing wearing of the opposing dentition. To obtain a smoother surface of the restoration, after milling, a polishing is necessary. This can be performed using different disks, polishing kits and pastes depending on the material or the operator’s preference. Finopol Diamond Polisher rubber wheels (Finopol, Praha, Czech Republic) or Hatho Habbras Discs (Hatho, Eschbach, Germany) are examples of utilized instruments. Glaze application, according to the manufacturer’s instructions, is a recommended procedure for ceramic CAD/CAM restorations. Afterwards, it is fired in a ceramic oven. Examples of glaze products are Vita Akzent Fluid and Vita Glaze AKZ 25, Empress Universal Glaze and IPS Empress Glaze Paste [116]. Glazing is also used to obtain the effect of advanced characterization. After the restoration is cleaned, dried and fixed, the operator can apply small amounts of shades and stains at the surface of the restoration. To obtain the desired colours and workable consistency, it is necessary to mix glaze liquid and different shades and stains on the pad. Glaze should be applied by a dedicated brush in a very subtle way, considering that the firing process results in a more intense appearance. It is recommended to apply a small amount of incisal shade to the incisal region and cusps in order to increase translucency. To give a restoration a more natural look, it is possible to apply warm colours to the area of the central fossa. After this characterization, short bursts of glaze spray in two or more series on the axial walls following the occlusal wall should be applied. Once a restoration is glazed and dried, the operator may proceed to the firing procedure using an appropriate programme. The subsequent steps are the cooling, cleaning and cementation of the restoration. The glazing procedure was summarised in the example of IPS e.max CAD Glaze given by the manufacturer Ivoclar Vivadent [117].

A study investigating the colour stability of zirconia-reinforced lithium silicate ceramic and lithium disilicate glass ceramic in beverages after two months showed that the glaze procedure led to enhanced colour stability. Moreover, authors noticed that due to the polishing, all changes in colour were clinically acceptable [113]. Another study on CAD/CAM materials’ surface roughness proves that mechanical polishing is capable of reducing the surface roughness, while glazing is facultative for fully crystallized/polymerized materials and desirable for partially crystallized materials, such as lithium-based ceramics to reduce the effect of roughening by milling [116]. The study by Tekçe N et al. on the surface of selected CAD/CAM resin restorative materials also results in the conclusion that glazing the surface contributes to its smoothing [118]. Similar conclusions were made by Vichi et al. in a study, where the roughness and gloss of the surface of VITA Suprinity and IPS e.max CAD (silica-based glass ceramics) were assessed after finishing and polishing. Authors claim that the most effective procedures for lowering the roughness and yielding the highest gloss of tested CAD-CAM materials are manual finishing and polishing for 60 s and applying glazing paste [119]. On the other hand, when glaze is not applied or the restoration requires additional adjustment in a patient’s oral cavity after the finishing procedure, faster plaque accumulation and discoloration may occur [119]. There is also a theory that finishing and polishing the surface of hybrid ceramic materials may negatively affect the physical properties of the restoration [120]. Marrelli et al. devote a lot of attention to surface roughness, which may influence the mechanical strength of zirconia-based CAD/CAM crowns and bridges. In their study, it is also proved that colouring using a commercial colouring liquid (Zirkon Zahn) has no significant effects on the mechanical strength of the zirconium ceramic-based specimens (the example of Kavo Everest Bio ZS Blank) [121].

## 14. Biocompatibility and Cytotoxicity of CAD/CAM Materials

It is extremely important for the new dental restoration to adapt to the conditions in the oral cavity, not only regarding their shape but with mechanical and physical properties. The crucial quality in the biological aspect is the compatibility with the surrounding tissues. Biocompatibility is an interdisciplinary phenomenon which covers biological, chemical and physical interactions and is highly connected with the concept of cytotoxicity—mainly in terms of the cellular response.

In addition to possessing the mechanical properties, as well as chemical and thermal resistance comparable to human bone, a CAD/CAM material needs to be biocompatible with the surrounding tissues. A material is expected not to cause any irritation, swelling or any kind of intolerance in the oral cavity. Therefore, a potential material needs to be evaluated in the scope of biocompatibility.

Human biofilm contains about 1000 species of bacteria [122] that adhere not only to the surface of the teeth but also to a prosthetic restoration. This adhesion depends on the type and roughness of the material’s surface. The adhesion and development of microorganisms being part of biofilm on different materials used for CAD/CAM was studied. As a result, it can be claimed that tested sintered materials, such as IPS e.max and polished IPS e.max, showed the best “anti-adhesive properties” with respect to *Streptococcus mutans* and *Lactobacillus rhamnosus* [99]. In another study, materials such as VITA CAD-, Celtra Duo, IPS e.max CAD and VITA YZ were tested to determine the cytotoxic effects and collagen type I secretions on human gingival fibroblasts. Results show that after 72 h, all groups reached biologically acceptable levels of cytotoxic potential. Moreover, it is concluded that the ceramic materials (lithium disilicate) present a better cell response than the polymers [123].

In terms of polymeric materials, the biocompatibility assessment of PEEK has been conducted since the early 1990s, and in vivo biocompatibility has been assessed as positive [124].

Acrylates are another vast branch of materials used in dentistry and specifically in CAD/CAM technology. They are known for their allergy potential. However, it is shown that due to the material processing, this effect can be minimized, and the general biocompatibility is sufficient for dental application. The aforementioned biocompatibility is confirmed by a very small and practically undetectable level of residual toxic monomer in the samples evaluated in vivo and in vitro tests [77].

Considering composites as a material for CAD/CAM blocks, there is a vital aspect of monomer release which depends on the degree of polymerization and further degradation. Depending on the degree of conversion and monomer composition, bisphenol dental composites can release bisphenol A (BPA; low weight monomers, such as HEMA and TEGDMA; high weight monomers, such as Bis-GMA and UDMA; and additives, such as free radicals and photoinitiator molecules) [98]. Composite blocks currently available for CAD/CAM technology are characterized by more applicable biocompatibility properties. They exhibit a higher degree of conversion, utilize less toxic monomers in their composition and lack in photoinitiators [98]. There are studies performed on RCB, Lava Ultimate (LAVA) and Vita Enamic (VITA) and Paradigm MZ100 (MZ100) apparatus, which compare resin blocks for CAD/CAM and conventional composites, which prove that no monomer elution is seen from the RCB. However, the authors stipulate that resin blocks for CAD/CAM show some worrisome results regarding cytotoxicity, and they require more studies [125].

Another evaluation of resin material was performed in regard to human gingival fibroblast response. Materials were divided into several groups with different chemical compositions and fabricating methods. CAD/CAM materials in the study were represented by Yamahachi dental materials containing poly(methyl methacrylate) polymer and a prefabricated hybrid ceramic block Vita Enamic, which is a polymer-infiltrated ceramic and was used as a control group. The results show that poly(methyl methacrylate) and bis-acryl have lower cytotoxicity to human gingival fibroblasts than poly(ethyl methacrylate). Moreover, CAD/CAM restorations, as they are prefabricated from resin blocks, prevent residual monomer and achieve high cell attachment [126]. Another study conducted on gingival cells proved no significant difference in CAD/CAM block cytotoxicity [78].

Studies on the biocompatibility of lithium metasilicate glass ceramics utilized in the CAD/CAM technique were performed. The material was not assessed as cytotoxic with the usage of methyl tetrazolium salt and Alizarin Red. It showed the best cellular adhesion and proliferation after 21 days [127].

## 15. Current Demand for the CAD/CAM Restorations

As already mentioned, CAD/CAM technology is constantly improving and gains popularity among dental offices and their patients. However, as an expensive and innovative technology, it is not available for everyone. Moreover, if the dentist does not perform a large volume of restorations, the CAD/CAM system investment will not pay off [39]. Most dental practices offering restorations made using CAD/CAM are located in high-income areas, such as Western Europe and the United States of America [41]. In 2016, more than 30,000 dentists around the world owned scanning and milling machines. They are most popular in the United States and Canada where almost one-third of all CAD/CAM devices are used. Moreover, all over the world, more than 15 million CEREC restorations alone have been completed [32]. In Poland, there are around 30 dental practices that use the CAD/CAM Cerec System [128]. At this point, it is worth mentioning that in Poland, the country of origin of this review, dental services provided using CAD/CAM technology are not part of the benefits reimbursed by the National Health Found. Remaining only at the field of private dentistry, they are available for the part of the society who can afford them. In many cases, it is not considered that the final effect depending on long-term clinical success and patients’ quality of life with this type of prosthetic restoration may outperform other clinical approaches. There are many clinical studies proving the long-term success of CAD/CAM restorations [108,129,130], which can confirm this thesis.

## 16. Conclusions

There is a wide range of materials used in CAD/CAM, including polymers, ceramics and composites, which are becoming more accessible and easier to handle. This review presented modern materials for CAD/CAM along with their characterization and highlighted their mechanical and clinical properties enabling satisfactory long-term restorations. Furthermore, high biocompatibility and aesthetic properties are subsequent advantages of the described material group.

New generations of powder-free intraoral scanners with greater resolution are being developed; hence, the restorations are designed faster and more accurately for a higher clinical success rate and prolonged longevity. The technology is gaining a strong position in dentistry, especially in the field of fixed partial dentures and crowns. Undeniably, the technology has its downsides, including the cost (of both materials and equipment) as well as the need for highly qualified personnel. Moreover, the correct selection of a material requires an experienced clinician. The technique of producing prosthetic restorations must always be adapted to every patient personally. For example, in the case of maxillo-mandibular relation disorders, the inability to define an occlusal plan may occur. Another limitation is the inaccurate horizontal and occlusal vertical dimension. Each clinical case has to be considered individually, and material and methods have to be chosen in order to meet the patient’s personal needs.

We can observe an increased number of composite materials on the market due to the possibility of mixing the properties of both the polymer, ceramic matrix and other filler particles. The comparison and selection of the correct type of material allows us to provide the patient with restorations characterized by acceptable and satisfying durability, biocompatibility and aesthetics. There are multiple mechanical and clinical parameters describing the materials, many of which are given by the manufacturer or are possible to find in the literature (e.g., provided in this article).

In the field of in situ restoration materials, there is still a necessity for advancement in terms of developing materials with superior properties to the contemporary used materials, as well as conducting long-term studies of the biocompatibility and wear of multiple materials in vivo. The aspect of surface finishing is of particular interest in the industry, as it is proved to affect the mechanical properties of the restoration. The application of CAD/CAM in dentistry provides state-of-art dental care. Hence, it is vital for the CAD/CAM framework in dentistry to be developed for further benefit of the patients.

## Figures and Tables

**Table 1:** Examples of commercial ceramic CAD/CAM material with its application.

Type of Ceramics	Brand, Manufacturer	Clinical Application
Resin Matrix Ceramics	Lava Ultimate, 3M-ESPE VITA Enamic, VITA-Zahnfabrik Cerasmart, GC	Onlays, inlays, veneers, single crowns, implant crowns
Lithium Silicate Ceramics	IPS e.max CAD, Ivoclar Vivadent VITA Suprinity PC, VITA Zahnfabrik Celtra Duo, Dentsply Sirona	Inlays, onlays, veneers, crowns
Leucite-Reinforced Glass Ceramics	IPS Empress CAD, Ivoclar Vivadent	Veneers, inlays, onlays, crowns
Zirconium Oxide Ceramics	NobelProcera Zirconia, Nobel Biocare	Single crowns, bridges, prosthetic restorations covering the entire dental arches, mainly posterior segment
Aluminium Oxide Ceramics	InCeram Alumina, VITA Zahnfabrik	Single crowns, bridges

## Data Availability

The data presented in this study are available in the article.
